# Type 1 Tympanoplasty Outcomes between Cartilage and Temporal Fascia Grafts: A Long-Term Retrospective Study

**DOI:** 10.3390/jcm11237000

**Published:** 2022-11-26

**Authors:** Salvatore Ferlito, Gianluca Fadda, Jerome Rene Lechien, Giovanni Cammaroto, Ricardo Bartel, Andrea Borello, Giovanni Cavallo, Francesca Piccinini, Ignazio La Mantia, Salvatore Cocuzza, Federico Merlino, Andrea Achena, Cristina Brucale, Quentin Mat, Stéphane Gargula, Nicolas Fakhry, Antonino Maniaci

**Affiliations:** 1 Department of Medical and Surgical Sciences and Advanced Technologies “GF Ingrassia” ENT Section, University of Catania, 95123 Catania, Italy; 2 Department of Otorhinolaryngology, San Luigi Gonzaga University Hospital, Universita degli Studi di Torino, 10121 Turin, Italy; 3 Department of Human Anatomy and Experimental Oncology, Faculty of Medicine, UMONS Research Institute for Health Sciences and Technology, University of Mons (UMons), 7000 Mons, Belgium; 4 Department of Otolaryngology-Head and Neck Surgery, Ambroise Paré Hospital (APHP), Paris Saclay University, 75016 Paris, France; 5 Department of Otolaryngology-Head and Neck Surgery, CHU de Bruxelles, CHU Saint-Pierre, School of Medicine, Université Libre de Bruxelles, 1000 Brussels, Belgium; 6 Department of Otolaryngology-Head and Neck Surgery, Foch Hospital, Paris Saclay University, 75016 Paris, France; 7 Department of Otolaryngology-Head and Neck Surgery, Morgagni Pierantoni Hospital, 47121 Forli, Italy; 8 Department of Otorhinolaryngology and Head and Neck Surgery, Hospital Universitario Mutua Terrasa, 8080 Barcelona, Spain; 9 U.O.C. di Otorinolaringoiatria ASST Grande Ospedale Metropolitano Niguarda, 20100 Milano, Italy; 10 Department of Medicine, Neurology, CHU de Charleroi, 15022 Charleroi, Belgium; 11 Department of Otorhinolaryngology, Lariboisière Hospital, Assistance Publique-Hôpitaux de Paris, 75016 Paris, France; 12 Service d’Otorhinolaryngologie et de Chirurgie Cervico-Faciale, Université Aix-Marseille, Hôpital de La Conception, 147, Boulevard Baille, 13005 Marseille, France

**Keywords:** tympanoplasty, myringoplasty, graft, cartilage, fascia, perforation

## Abstract

Background: To compare the functional and anatomical results of two different types of grafts in type 1 tympanoplasty (TPL I). Methods: A retrospective comparative bicentric study was conducted on patients treated with TPL I using temporal fascia or tragal cartilage. We evaluated the functional and anatomical results with intergroup and intragroup analyses. Variables predicting long-term success were also evaluated. Results: A total of 142 patients (98 fascia graft vs. 44 cartilage) were initially assessed, with a mean follow-up of 67.1 ± 3.2 months. No significant differences were observed between the two groups on the intergroup analysis of age, gender, ear side, or pre-operative hearing data (all *p* > 0.05). At the intragroup analysis of auditory outcomes, both groups demonstrated a significant improvement in post-operative air conduction, with greater gain for the fascia group at 6 months follow-up (*p* < 0.001 for both); however, at long-term follow-up, cartilage demonstrated better stability results (*p* < 0.001). When comparing the pre-and post-operative air-bone-gap (ABG), both groups showed a significant gain (*p* < 0.001); the fascia group showed that at 6 months, a greater ABG increase was found, but the difference was not statistically significant (4.9 ± 0.9 dB vs. 5.3 ± 1.2 dB; *p* = 0.04). On the contrary, the cartilage group at long-term follow-up at 5 years maintained greater outcomes (10 ± 1.6 dB vs. 6.4 ± 2 dB; *p* < 0.001). Lower age (F = 4.591; *p* = 0.036) and higher size of perforation (F = 4.820; *p* = 0.030) were predictors of long-term functional success. Conclusions: The graft material selection should consider several factors influencing the surgical outcome. At long-term follow-up, the use of a cartilage graft could result in more stable audiological outcomes, especially in younger patients or in case of wider perforations.

## 1. Introduction

Type I tympanoplasty, first classified by Zoller and Wullstein et al., is one of the most common surgical techniques performed to reconstruct the tympanic membrane (TM) [1,2]. The main goals are twofold: to restore the integrity and cleanliness of the middle ear cavity and to improve hearing. Among the various available graft materials, the choice falls mainly on temporalis fascia or cartilage, which have been extensively studied and have shown better results than other grafts, such as periosteum, perichondria, vein, and fat [3,4]. Several technical modifications have improved surgical outcomes in recent decades, but two basic grafting techniques have emerged, the overlay technique and the underlay technique [5,6,7]. Graft take-up rates have been estimated between 82% and 97% regardless of the technique used [6,8,9,10]. Conflicting data are reported in the literature regarding the association between tympanoplasty success rates and the size or site of the perforation [6,10,11,12,13,14,15].

Sözen et al. analyzed the use of cartilage grafts in patients with a higher probability of failure, comparing anatomical and functional outcomes with temporalis fascia grafts [15]. The cartilage graft minimized the inflammatory reaction of the tissue and provided great support against retraction, demonstrating a perforation closure ratio of 88.5% vs. 80.6%, of the fascia and the average ABG increase of 9.7 dB vs. 5.7, respectively.

Long-term results show success rates close to 90% [5]. Surgeon experience is a significant factor in tympanoplasty success rates [14].

Temporal fascia (TF) is abundant, transparent, thin and easier to harvest, but it lacks elasticity, a characteristic that makes it much more susceptible to pressure changes [8,13]. However, situations such as eustachian tube dysfunction, adhesive otitis media, and large perforations may predispose to therapeutic failure of TF and reduced results in long-term follow-up. In addition, the newly formed drum tends to retract [14]. This tendency has prompted the development of tympanic membrane cartilage grafting [15]. The most accessible site is the tragus. On the other hand, cartilage graft is stiffer than fascia, which makes it more resistant to infection, resorption, and retraction [3,16,17,18,19]. Several authors have conducted comparative studies on the two graft materials to evaluate their respective strengths and weaknesses [4,18,19,20,21,22,23,24,25,26,27].

An interesting systematic review by Mohamad et al. compared the effectiveness of using cartilage with temporalis fascia used in tympanoplasty [23]. The authors retrieved 3 RCTs, 11 observational studies and 4 evidence-based with a total of 1,475 patients. Although 4 studies reported more intact eardrum in the cartilage graft, no statistical difference was found in auditory outcomes vs. fascia graft.

Vashishth et al. in 2014 compared the functional and anatomical outcomes of 90 patients, 30 of whom were treated with cartilage and 60 with fascia [28]. Patients with fascia reported a significantly lower mean ABG gain (dB HL) than cartilage graft (13.2 ± 6.6 dB vs. 21.7 ± 6.7 dB; *p* < 0.001). However, a lower but not significant take rate was shown for the fascia (50/60 vs. 27/30 patients; *p* = 0.814). An interesting meta-analysis comparing cartilage and fascia in TPL I to pooled analysis showed no significant differences for auditory outcomes [29]. More specifically, the subgroup of full-thickness cartilage grafts had better auditory outcomes in the subgroup analysis than the temporalis fascia grafts. These studies showed an overall success rate of approximately similar morphological and audiological outcomes between cartilage and temporalis fascia [25]. In general, cartilage grafting is more effective morphologically or anatomically, but its rigidity is believed to have a negative impact on postoperative hearing levels [23,24].

Demirci et al. in 2015 compared the anatomical and functional outcomes of different graft materials used in pediatric tympanoplasty, considering an intact graft and an air-bone gap (ABG) ≤ 20 a surgical success in the postoperative period [25]. The authors found a better graft success rate in cartilage vs. fascia (92% vs. 82.9%; *p* < 0.001). Yet, the authors demonstrated no significant differences between the fascia and cartilage groups for either hearing enhancement or graft success rate.

In this regard, we conducted a retrospective comparative analysis to evaluate the long-term postoperative results of cartilage and temporalis fascia grafts by analyzing the anatomic-auditory outcomes.

## 2. Materials and Methods

A retrospective bicentric study was performed on 142 patients diagnosed with chronic otitis media (COM) who underwent a TPL I between October 2010 and July 2016.

**Figure 1:**
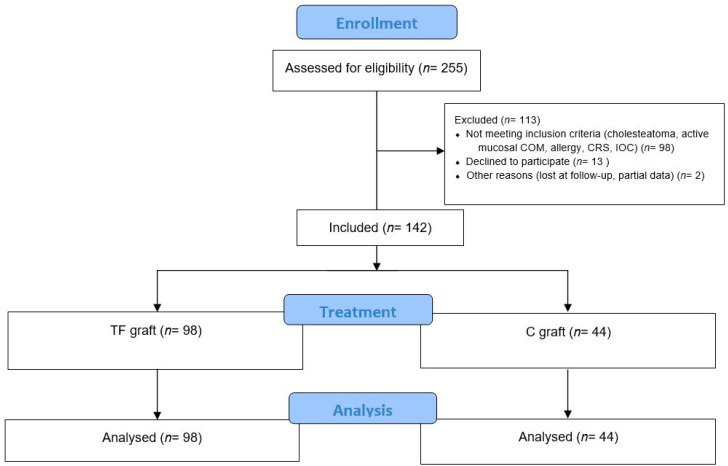
CONSORT 2010 Flow Diagram. Abbreviation: TF, temporalis fascia; C, Cartilage; COM, chronic otitis media; CRS, chronic rhinosinusitis; IOC, Impaired ossicular chain.

Consequently, all patients were retrospectively analyzed until 1 July 2022 to reach an average follow-up of 5 years. Patients were enrolled in two different medical centers. The patient selection process is summarized in Figure 1.

Patient features and demographic data such as age, sex, localization and perforation, type of graft material used, relapse, pre-operative and post-operative hearing levels were recorded (Table 1).

### 2.1. Operative Techniques and Follow-Up

**Figure 2:**
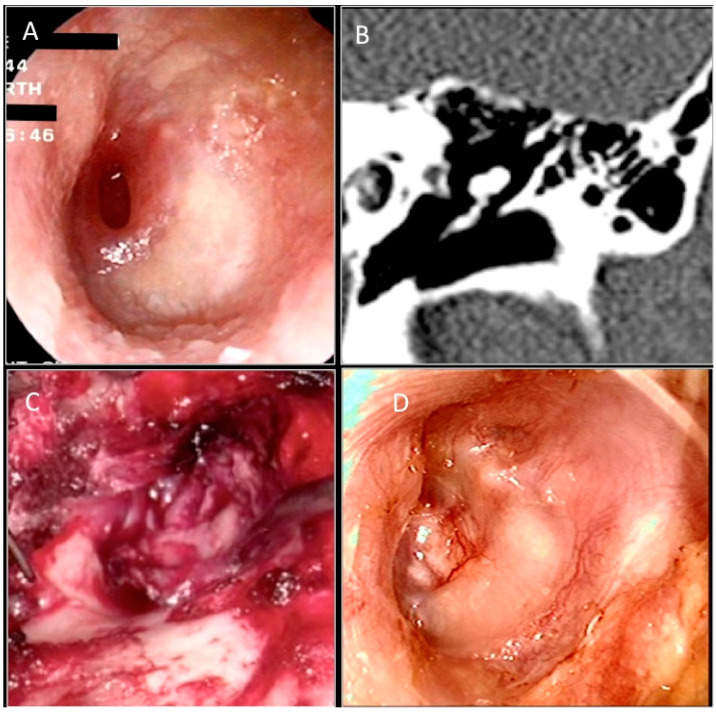
(**A**) Preoperative otoendoscopic image showing anterior perforation of the left tympanic membrane. Anterior wall of the procident duct. (**B**) CT scan in coronal section. (**C**) Intraoperative image of temporalis muscle fascia placed with Underlay technique. (**D**) Otoendoscopic follow-up at 6 months.

Two expert otosurgeons working in two different otologic centers performed all operations using an operative microscope (Zeiss Surgical Microscope OPMI) under general anesthesia. All cases were executed using the postauricular approach with the underlay technique. The postauricular incision was made about 3 mm behind the postauricular area to expose the temporalis fascia in the fascia group (Figure 2). A larger piece of loose areolar temporalis fascia is removed, pressed, and dried under a heat lamp (approximately 2.5 × 1.5 cm).

**Figure 3:**
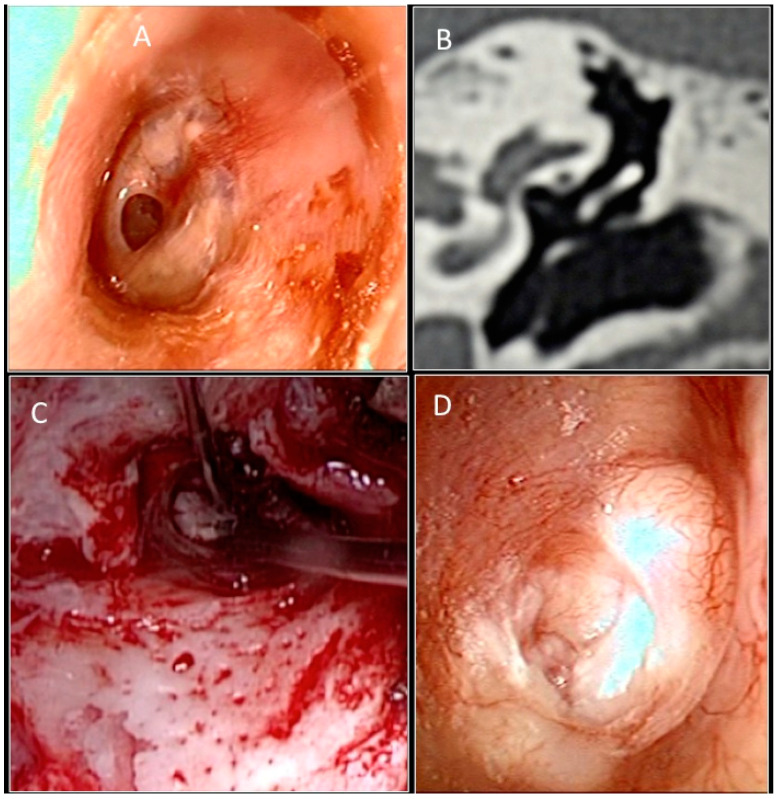
(**A**) Preoperative otoendoscopic image showing anterior perforation of the left tympanic membrane and signs of tympanosclerosis. (**B**) CT scan in coronal section. (**C**) Intraoperative image of molded tragal cartilage placed with Underlay technique. (**D**) Otoendoscopic follow-up at 6 months.

Subsequently, a T-shaped incision is made through the subcutaneous tissue in order to expose the periosteum of the mastoid bone, the temporal line, and the external auditory canal. The vascular strip is lifted out of the external auditory canal and placed under the anterior blade of a self- retaining retractor. The inferior flap is elevated to the fibrous annulus. The graft was then placed under the tympanic remains. Absorbable gelatin sponge (Spongostan) is packed into the middle ear cavity to lie under the annulus anteriorly. The graft is held securely in position when the inferior and superior flaps are replaced. The ear canal is filled with an absorbable gelatin sponge. In the tragal cartilage group, an incision was made 1 mm on the medial side of the tragus and carried through the perichondrium and tragal cartilage (Figure 3).

This leaves cartilage remaining at the tip of the tragus to maintain its shape. A piece of tragal cartilage with perichondrium is removed using small-pointed scissors. The perichondrium is reflected from the surface of the cartilage, similar to a book cover, and the cartilage is trimmed to size. The perichondrium is placed anteriorly under the malleus and onto posterior canal wall.

A mastoid dressing was applied for the first 24 h postoperatively. The patient was placed on topical antibiotics (Ciprofloxacin) to soften the Spongostan in the external auditory canal from the second week. Follow-up controls were scheduled at the 1st, 3rd, 6th, and 12th months; after that, the patient was seen at yearly intervals to obtain an average follow-up of 5 years. Pure tone audiometry (PTA) was used preoperatively and at each control to evaluate the functional result of TPL I. The threshold was established for 0.5, 1, 2, and 4 kHz frequencies. Air-bone gap (ABG) was calculated as the average difference between the air conduction and bone conduction at 0.5, 1, 2, and 4 kHz. We considered an ABG ≤ 20 dB as functional success, while anatomical success was defined as intact graft without perforation, retraction, lateralization, and dry status. Time and localization of relapse were evaluated in both groups.

### 2.2. Statistical Analysis

Data analysis was performed using IBM SPSS Statistics for Windows, IBM Corp. Released 2017, Version 25.0. Armonk, NY: IBM Corp. Descriptive statistics were reported on average ± standard deviation or proportion. Data normality was assessed using the Kolmogorov–Smirnov test of normality. The *t*-test for paired samples was used to determine the difference between observations. The Mann–Whitney U test was performed to analyze group differences. The tests were two-tailed, and a *p*-value of <0.05 was considered statistically significant.

## 3. Results

A total of 142 patients, 70 males and 72 females, were enrolled in this study. The average age was 53.3 ± 17.6 years. The mean duration of follow-up was 67.1 ± 3.2 months in the fascia group and 66.6 ± 2.5 months in the cartilage graft group; the difference was insignificant (*p* = 0.36)(Table 1).

Of the 142 subjects, 98 were enrolled in the fascia graft group, while 44 were in the cartilage group. We did not find significant differences among the groups regarding age, gender, ear side, or pre-operative hearing data (*p* > 0.05 for all) (Table 1 and Table 2). The groups differed for perforation size (>50% vs. <50%; *p* < 0.001). A higher recurrence rate was found in the fascia graft group (15/98, 15.3% vs. 4/44, 9.09%; *p* = 0.37).

All reperforations reported at follow-up were <50% in size and located in the anterior quadrant.

At intragroup analysis for 6 months hearing outcomes, both groups demonstrated a significant improvement in post-operative air conduction from baseline, with greater gain for the fascia group (10.2 ± 1.9 dB vs. 11.4 ± 1.0 dB; *p* < 0.001) than cartilage (Table 2).

The average post-operative bone conduction was better for the fascial group (5.3 ± 1.7 dB vs. 6.1 ± 2 dB; *p* < 0.001).

As regards pre-and post-operative ABG, both groups showed a significant gain (*p* < 0.001 for both); at 6 months control, the comparison was not significant (4.9 ± 0.9 dB vs. 5.3 ± 1.2 dB; *p* = 0.04); however, the cartilage group showed a higher mean ABG gain stability at long-term follow-up (6.4 ± 2.0 dB vs. 10.0 ± 1.7 dB; *p* < 0.001).

The functional success rates (post-operative ABG < 20 dB) at 5 years were 66.81% (25/44) in the fascia group and 75.81% (74/98) in the cartilage graft group (*p* = 0.33).

At the sub analysis of hearing levels at 5 years according to the presence of reperforation or not, worse hearing levels were found at mean AC in the group with reperforation for both fascia graft and cartilage (*p* < 0.001) (Table 3).

The overall multivariate analysis demonstrated as independent factors worsening the 5-year functional success age (F = 4.591; *p* = 0.036) and larger perforation (F = 4.820; *p* = 0.030) (see Table 4).

## 4. Discussion

The primary goal of tympanoplasty is to repair the tympanic membrane to restore membrane integrity, prevent contamination from pathogen exposure, and restore the vibratory area of the membrane, improving auditory performance [18,19,30,31].

Anatomical and functional outcomes are influenced by multiple variables, such as the location and size of the perforation, chronic respiratory disorders with adhesive or bilateral ear infections, or revision surgery [32,33,34,35]. Therefore, the type of graft used must take into account the properties of each material, the patient’s general condition, and the possibility of graft integration. Temporal muscle fascia and tragus cartilage are the most frequently used materials for TPL I in the literature because of the ease of graft harvesting and high surgical success rate [22,23,25]. However, the superiority of one graft over the other has long been debated [4,17,18,19].

The dissatisfaction with fascia temporalis is correlated with the higher incidence of recurrent reperforation and retractions, predominantly due to the lower strength and stiffness of the graft [19,20,21,22,23,24,25]. The temporal fascia consists of a strong fibrous sheet divided into clearly distinguishable superficial and deep layers that originate from the same region with the muscle fibers. Such characteristics and physical properties especially due the arrangement of the elastic collagen fibers of the material gives it better absorption and transmission of the sound wave than other types of grafts. Yet, such malleability and elasticity predispose it to lower resistance in case of larger caliber perforations or tubal pathologies. In contrast, in the case of recurrence or wider perforations the use tougher material should be indicated. Tragal cartilage is in fact made up of a specialized supporting connective tissue, in which cells called chondrocytes are immersed in an abundant amorphous intercellular substance, synthesized by themselves, consisting of collagen fibers and a gelatinous amorphous matrix [14,15,16,17,18]. This scaffold gives it the main characteristics such as solidity, flexibility although less than the temporalis fascia, and the ability to deform limitedly.

The role of the physical properties of the temporalis fascia and cartilage on functional outcomes as well as how graft preservation methods affect physical and chemical properties preserved at low temperatures are discussed in the literature [36,37]

Trindade et al. examined temporalis fascia tensile strength, noting how patient age significantly affects audiological outcomes especially in older subjects [37]

As reported by Tarumoto et al. al comparison between the results of cold storage and fresh retrievals did not occur a significant difference in temporal band strength [36].

Yüksel Aslıer et al. in a study of 70 ears analyzed the differences of pure-tone audiometry thresholds and wideband ambient-pressure absorbance ratios with respect to the graft material, graft thickness, cartilage surface area ratio and elapsed time after surgery were analyzed [38]. The authors found energy absorbance ratios at 2000 and 2828 Hz frequencies higher in cartilage grafts patients vs. control while common effects of independent variables at 8000 Hz.

Onal et al. compared in this regard the functional outcomes of patients with bilateral COM and subtotal perforation treated with TPL I with temporalis fascia (*n* = 41) or cartilage graft (*n* = 39) [39]. TPL I with cartilage in these subjects demonstrated better auditory outcomes (23 ± 8.4 dB vs. 28.5 ± 14.2 dB; *p* < 0.001) and graft success rates than fascia (92.3% vs. 65.9%; *p* < 0.001). However, the authors treated only subtotal perforations, and a comparison of early and long-term outcomes was not performed.

In contrast, Demirpehlivan et al. reported similar postoperative results in terms of air conduction gain between the two graft types (24.54 dB cartilage vs. 24.51 dB fascia; *p* > 0.05), emphasizing the superior take rate of the cartilage graft (97.06% vs. 80.6%; *p* < 0.001) [19]. In our study, however, the highest recurrence rate was found in the fascia graft group, although not significant (15/98, 15.3% vs. 4/44, 2.27%; *p* = 0.37). In terms of auditory outcomes, on the other hand, both groups demonstrated significant improvement in postoperative air conduction, although a greater gain was found in the fascia group (*p* < 0.001). However, our results, which report better short-term results for the fascia graft but greater long-term stability for the cartilage, have an important limitation due to the small sample size. Currently, cartilage graft is believed to show a higher rate of graft than TF. Jalali et al. in this regard included 11 prospective and 26 retrospective studies with a total of 3,606 patients [40]. Indeed, the authors showed cartilage graft demonstrated better outcomes than fascia graft in a pooled analysis of overall TPL I graft integration rates (92% vs. 82%; *p* < 0.001). In addition, although there were no significant differences in ABG between the two groups, the subanalysis demonstrated better outcomes for patients in the temporalis fascia group (*p* = 0.02).

Cartilage TPL I offers better results in patients bilateral perforation, revision surgery, or otorrhea at the time of surgery, probably due to its greater mechanical stability than fascial grafting [16,18]. Due to its low metabolic rate, which facilitates graft integration, cartilage is particularly effective in the presence of negative pressure. Long-term outcomes are an important issue for integration and functional outcomes postoperatively [13,19,25].

Yung et al. compared 38 patients with tympanic perforation in a randomized clinical trial, including 20 fascial grafts and 18 cartilage grafts, with a mean follow-up of 24 months [22]. The authors found no statistically significant differences at 24 months in either recovery rate (cartilage 16/20, 84.2% vs. fascia 12/18, 80%; *p* = 0.716) or postoperative ABG (16.9 ± 10.1 dB vs. 20.6 ± 8.1 dB; *p* = 0.230). However, a limitation of the study is the small sample size of enrolled patients.

Long-term auditory outcomes are debated in the literature, especially when comparing the two different graft types.

Yetiser et al., in a comparative study of 113 patients and a mean follow-up of 3.2 years, reported a significantly better postoperative air-bone gap in the cartilage group (14.2 ± 7.7 dB vs. 19.7 ± 12.0 dB in the fascia group; *p* = 0.008) [18].

On the other hand, the randomized controlled trial conducted by Cabra et al. on 123 patients with COM found better morphologic success at 24 months in the cartilage group than in the fascia group (82.26% vs. 64.41%; *p* = 0.03) [16]. In addition, the relative risk of morphologic success was 1.36 (95% CI = 1.11–1.38).

Our study proposed larger long-term results than previous studies, providing more information about the stability of the results of the two methods.

When comparing air gap levels at baseline vs. long-term, a significant gain was found in both groups (*p* < 0.001 for all); however, at subgroup analysis, the cartilage group showed significantly greater improvements than the fascia (6.4 ± 2.0 dB vs. 10.0 ± 1.7 dB; *p* < 0.001).

Several predictors of audiological and anatomical success have been described in the literature as possible dependent variables, such as the location of the perforation and the extent of postoperative inflammation [28,29,30,31,32,33,34,35,36,37,38,39,40].

Al Lackany et al. reported that auditory outcomes were better in central perforations in the fascia group than in the cartilage group, while larger perforations (subtotal or total) were more successful in the cartilage group [41].

In contrast, the multivariate analysis conducted in our study showed that the independent factors influencing functional success at 5 years were age (F = 4.591; *p* = 0.036) and larger perforation (F = 4.820; *p* = 0.030).

However, a relevant limitation of our study is that the ratio of perforations <50% and >50% in size was unbalanced, probably due to the fact that smaller perforations were treated more with fascia graft.

## 5. Conclusions

At early follow-up, improved audiological results might be obtained by using a fascia graft, especially in younger patients or those with smaller perforations. However, more stable postoperative outcomes are provided by cartilage graft, with significant maintenance of auditory function and lower long-term rates of reperforation.

## Figures and Tables

**Table 1:** The main demographic features of both groups included.

	Fascia Group (*n* = 98)		Cartilage Group (*n* = 44)		
	*n*/SD	%	*n*/SD	%	*p*-Value
Age (year)	52.5 ± 18.9		55.3 ± 14.5		0.38
Mean follow-up (months)	67.1 ± 3.2		66.6 ± 2.5		0.36
Gender					
Male	46/98	46.93	24/44	54.54	0.40
Female	52/98	53.06	20/44	45.46	
Side					
Left	54/98	55.1	27/44	61.36	0.48
Right	44/98	44.9	17/44	38.64	
Perforation size					
<50%	49/98	50	8/44	18.18	<0.001
>50%	49/98	50	36/44	81.72	
Anterior	16/98	16.32	11/44	25	0.83
Posterior	33/98	33.67	25/44	75	
Recurrence rate	15/98	15.30	4/44	9.09	0.37

Patients with cholesteatoma, active mucosal COM, allergy, chronic rhinosinusitis, an age younger than 10 years and older than 70 years, and an impaired ossicular chain were excluded. Thus, the participants were divided into two groups according to the material graft used, temporalis fascia or tragal cartilage, and functional and anatomical results were compared.

**Table 2:** Hearing outcomes comparison between early and long-term follow-up.

	Fascia Group (*n* = 98)	Cartilage Group (*n* = 44)	*p*-Value
Air conductive (AC) (dB)			
Pre-operative	24.6 ± 6.81	26.58 ± 7.81	0.128
500	35.2 ± 4.47	34.24 ± 4.01	
1000	34.73 ± 4.18	30.17 ± 3.15	
2000	20.9 ± 3.98	25.6 ± 3.74	
4000	6.21 ± 2.87	15.61 ± 2.23	
Post-operative 6 months	11.23 ± 3.92	11.54 ± 4.18	<0.001
500	14.78 ± 3.23	15.75 ±2.93	
1000	10.97 ± 3.46	9.78 ± 3.64	
2000	11.25 ± 3.49	10.18 ± 4.15	
4000	6.7 ± 2.5	5.87 ± 3.11	
Post-operative 5 years	15.32 ± 4.39	11.68 ± 4.78	<0.001
500	21.2 ± 4.68	14.56 ± 3.48	
1000	20.3 ± 3.69	14.98 ± 4.83	
2000	10.86 ± 3.16	11.38 ± 3.81	
4000	10.27 ± 3.95	5.13 ± 4.12	
Bone conductive (BC)	5.73 ± 1.23	5.31 ± 0.95	0.46
500	5.81 ± 2.9	5.35 ± 3.28	
1000	5.32 ± 4.33	5.81 ± 3.42	
2000	5.08 ± 1.58	5.02 ± 2.85	
4000	4.93 ± 2.58	4.71 ± 2.63	
ABG (dB)			
Pre-operative	20.3 ± 3.0	19.3 ± 2.7	0.04
Post-operative 6 months	4.9 ± 0.9	5.3 ± 1.2	0.04
Post-operative 5 years	10.0 ± 1.7	6.4 ± 2.0	<0.001

**Table 3:** 5 years subgroup analysis according to reperforation among the two groups enrolled.

	Fascia Group (*n* = 98)		*p*-Value	Cartilage Group (*n* = 44)		*p*-Value
	Perforated	Non-Perforated		Perforated	Non-Perforated	
Air conductive (AC) (dB)	20.9 ± 2.4	15.4 ± 1.7	<0.001	15.7 ± 1.3	12.3 ± 1.0	<0.001
Bone conductive (BC)	10.2 ± 1.8	5.2 ± 1.5	<0.001	8.3 ± 2.4	5.8 ± 2.2	<0.001
ABG (dB)	10.5 ± 2.1	10.1 ± 1.7	0.002	7.3 ± 2	5.1 ± 1.5	0.01

**Table 4:** Multivariate analysis of prognostic factors for long-term functional success in the total sample.

Source Dependent Variables		Mean Square	F	Sig.
Functional success	Sex	0.041	0.161	0.689
	Age	1.107	4.591	0.036
	Side	0.029	0.118	0.733
	Perforation type/size			
	>50%	1.148	4.820	0.030
	<50%	0.452	1.821	0.179
	Anterior	0.160	1.153	0.287
	Posterior	0.002	0.011	0.916
	Graft type	0.257	1.239	0.269

## Data Availability

Not applicable.
